# Is the Evaluation of Robot-Assisted Surgery Based on Sufficient Scientific Evidence?

**DOI:** 10.3390/jcm12020422

**Published:** 2023-01-04

**Authors:** Alain Bernard

**Affiliations:** Department of Thoracic and Cardiovascular Surgery, Dijon University Hospital, 21079 Dijon, France; alain.bernard@chu-dijon.fr

Robot-assisted surgery is becoming an increasingly common approach for lung cancer resection. This technological innovation is the subject of numerous publications, which demonstrates the interest in this approach. All these publications report contradictory results on the clinical benefits of robot-assisted surgery.

Considering the large number of publications, the quality of the studies raises questions. We are surprised by the small number of randomized controlled trials (RCTs) on robot-assisted surgery. We found three RCTs, one of which is currently being included, while the other two have published their results with small numbers, 113 and 76 patients [1,2,3]. In contrast, many publications are based on non-randomized observational data.

The WHO publication on the assessment of new technologies recalls the importance of evidence-based studies [4]. Not only do they help to inform decision makers, but they are also necessary for patients as an opportunity to confirm therapeutic innovation. Best practice studies protect patients by explicitly informing them about the aims of the study and possible complications. An evidence-based study minimizes bias to avoid drawing wrong conclusions based on non-existing data. A convincing study is one with the highest level of evidence [4]. The only design that meets these requirements is a randomized controlled trial (RCT). In this design, by randomly assigning one of the treatments to patients, the two groups of patients are made comparable, as only the assigned treatments differ. It is the only design that allows one treatment to be found superior to the other.

We regret the lack of enthusiasm of thoracic surgeons to participate in RCTs to demonstrate the value of robot-assisted surgery. We submitted an RCT for publication comparing VATS with thoracotomy. We had great difficulty in completing this trial, due to the delay in inclusion because of the lack of motivation on the part of surgeons to include patients. In the last ten years, we identified a total of only four RCTs published on VATS [5,6,7,8]. At the same time, we observed a multitude of publications using databases where several thousand patients have benefited from the technology [9]. One or more large-scale RCTs would have been possible, as minimally invasive surgery currently represents 50% of the approaches to lung cancer surgery [10]. The lack of studies using the highest level of evidence will continue to cast doubt on the true superiority of minimally invasive surgery over thoracotomy.

The use of a propensity score applied to an observational cohort helps to limit bias. We can never be sure that these methods control for all confounding factors and that the two groups of patients are truly comparable. The clinical databases used in studies raise the question of data quality. One study showed that only 25% of the surgical teams participating in the national Epithor database included all their lung cancer patients and all postoperative deaths [11]. This finding suggests caution about the conclusions of studies using clinical databases. A study carried out in the United States using an observational cohort showed no difference in 5-year survival for robot-assisted surgery, compared with thoracotomy and VATS [12]. Another French study using the Epithor database reported poorer 5-year survival for patients who underwent robot-assisted surgery than for patients who underwent VATS [13]. What conclusion can be drawn about robot-assisted surgery with conflicting results from potentially biased studies?

Despite the widespread use of real-world evidence (RWE) and the use of methodologies to reduce bias, randomized controlled trials remain the main study design with the highest level of evidence to validate an innovative technology. RWE has its place following an RCT to confirm the results or highlight adverse events. We find it difficult to understand the lack of motivation of thoracic surgeons to participate in RCTs when other disciplines have been able to conduct large-scale RCTs to validate innovative technologies.

In conclusion, the lack of convincing studies to validate robot-assisted surgery will continue to cast doubts on the real benefit of this approach, particularly for decision makers or evaluation agencies. Robot-assisted surgery is thus penalized if it is to be considered a technology that can transform the management of patients with lung cancer.
